# Cycling hypoxia induces chemoresistance through the activation of reactive oxygen species-mediated B-cell lymphoma extra-long pathway in glioblastoma multiforme

**DOI:** 10.1186/s12967-015-0758-8

**Published:** 2015-12-28

**Authors:** Wei-Ling Chen, Chi-Chung Wang, Yu-Jung Lin, Chung-Pu Wu, Chia-Hung Hsieh

**Affiliations:** Aging Medicine Program, China Medical University, Taichung, Taiwan; Department of Psychiatry, Taichung Veterans General Hospital, Taichung, Taiwan; Graduate Institute of Basic Medicine, Fu Jen Catholic University, Taipei, Taiwan; Graduate Institute of Basic Medical Science, China Medical University, Taichung, Taiwan; Department of Physiology and Pharmacology, Chang Gung University, Tao-Yuan, Taiwan; Department of Medical Research, China Medical University Hospital, Taichung, Taiwan; Department of Biomedical Informatics, Asia University, Taichung, Taiwan

**Keywords:** B-cell lymphoma extra-long, Cycling hypoxia, Glioblastoma, Nuclear factor-κb, Reactive oxygen species

## Abstract

**Background:**

Cycling hypoxia is a well-recognized phenomenon within animal and human solid tumors. It contributes to the resistance to cytotoxic therapies through anti-apoptotic effects. However, the mechanism underlying cycling hypoxia-mediated anti-apoptosis remains unclear.

**Methods:**

Reactive oxygen species (ROS) production, activation of the hypoxia-inducible factor-1 alpha (HIF-1α) and nuclear factor-κB (NF-κB) signaling pathways, B-cell lymphoma extra-long (Bcl-xL) expression, caspase activation, and apoptosis in in vitro hypoxic stress-treated glioblastoma cells or tumor hypoxic cells derived from human glioblastoma xenografts were determined by in vitro ROS analysis, reporter assay, western blotting analysis, quantitative real-time PCR, caspase-3 activity assay, and annexin V staining assay, respectively. Tempol, a membrane-permeable radical scavenger, Bcl-xL knockdown, and specific inhibitors of HIF-1α and NF-κB were utilized to explore the mechanisms of cycling hypoxia-mediated resistance to temozolomide (TMZ) in vitro and in vivo and to identify potential therapeutic targets.

**Results:**

Bcl-xL expression and anti-apoptotic effects were upregulated under cycling hypoxia in glioblastoma cells concomitantly with decreased responses to TMZ through ROS-mediated HIF-1α and NF-κB activation. Tempol, YC-1 (HIF-1 inhibitor), and Bay 11-7082 (NF-κB inhibitor) suppressed the cycling hypoxia-mediated Bcl-xL induction in vitro and in vivo. Bcl-xL knockdown and Tempol treatment inhibited cycling hypoxia-induced chemoresistance. Moreover, Tempol treatment of intracerebral glioblastoma-bearing mice combined with TMZ chemotherapy synergistically suppressed tumor growth and increased survival rate.

**Conclusions:**

Cycling hypoxia-induced Bcl-xL expression via ROS-mediated HIF-1α and NF-κB activation plays an important role in the tumor microenvironment-promoted anti-apoptosis and chemoresistance in glioblastoma. Thus, ROS blockage may be an attractive therapeutic strategy for tumor microenvironment-induced chemoresistance.

## Background

Hypoxia is well evidenced within most solid tumors [1]. Acute, intermittent, and cycling hypoxia are associated with inadequate blood flow, whereas chronic hypoxia is the consequence of increased oxygen diffusion distance, resulting from tumor expansion [2]. These hypoxic areas can either promote cell death or provoke an adaptive response, leading to the selection for death resistance [3–5]. Once tumor cells adapt to hypoxia, they are more resistant to apoptosis and less responsive to cancer therapy. We recently showed that cycling hypoxia and chronic hypoxia are important tumor microenvironment phenomena that limit tumor response to chemotherapy in glioblastoma multiforme (GBM) [6]. Therefore, several potential mechanisms, including the lack of oxygen that is available for anti-tumor drugs to act, DNA over-replication, increased genetic instability, the anti-proliferative effect of hypoxia [7], increased multidrug resistance (MDR) linked to adenine triphosphate (ATP)-binding cassette (ABC) transporter [8, 9], are thought to play a role in cycling hypoxia-induced chemoresistance. However, the exact mechanisms triggered by cycling hypoxia, leading to resistance to apoptosis, remain unclear.

Previous studies indicated that the repetitive periods of hypoxia and reoxygenation could lead to an increased production of reactive oxygen species (ROS) [10, 11]. Moreover, NADPH oxidase subunit 4 (Nox4) is a critical mediator involved in cycling hypoxia-mediated ROS production, tumor progression, and resistance to cytotoxic therapies in GBM [12–14]. Although ROS play an important role in apoptosis induction under both physiologic and pathologic conditions, ROS have also anti-apoptotic effects in cancer cells through unknown mechanisms [15, 16]. A recent study reported that ROS stimulates NF-κB signaling, resulting in a B-cell lymphoma extra-long (Bcl-xL)-mediated resistance to drug-induced cell death [17]. Bcl-xL is a member of the Bcl-2 family of proteins and acts as a pro-survival protein by preventing the release of mitochondrial contents and caspase activation [18]. NF-κB can bind directly to Bcl-xL promoter to regulate its expression [19]. Furthermore, Bcl-xL is also one of hypoxia-inducible factor-1 (HIF-1) target genes because its promoter contains a hypoxia-responsive element (HRE) [20]. These observations point to the possibility that Bcl-xL is a critical contributor to cycling hypoxia-mediated anti-apoptosis and resistance to cytotoxic therapies.

In the present study, we determined the impact and mechanism of cycling hypoxia on anti-apoptosis and chemoresistance in GBM. Our results show that cycling hypoxic stress significantly increases resistance to temozolomide (TMZ) via Bcl-xL upregulation. ROS-mediated HIF-1α and NF-κB activation plays an essential role in cycling hypoxia-mediated Bcl-xL induction. Moreover, pretreatment with a ROS scavenger, Tempol, in intracerebral glioblastoma-bearing mice demonstrated a synergistic suppression of tumor growth and increased survival rate in TMZ chemotherapy, suggesting that ROS blockade before drug administration and in combination with chemotherapy may be an effective approach to suppress tumor microenvironment-induced chemoresistance and further improve the efficacy of chemotherapy in GBM.

## Methods

### Cell culture

U251, U87 and GBM8401 glioblastoma cells were cultured in DMEM (Life Technologies) supplemented with 10 % fetal bovine serum (FBS), 10 mM HEPES, and 1 % penicillin–streptomycin.

### In vitro hypoxic treatments

Cells were treated in Biospherix C-Chamber (Biospherix) inside a standard culture chamber by means of exhausting and gassing with 95 % N_2_ and 5 % CO_2_ to produce oxygen concentrations of 0.5–1 % at 37 °C to achieve hypoxic condition. Cells were treated with or without in vitro non-interrupted hypoxic or cycling hypoxic stress as previously described [6]. Briefly, cell cultures were exposed to 3 cycles consisting of 0.5–1 % O_2_ for 1 h interrupted by 5 % CO_2_ and air for 30 min for cycling hypoxic treatment and to persistent 0.5–1 % O_2_ for 4 h for non-interrupted hypoxic treatment.

### Real-time quantitative PCR (Q-PCR)

Q-PCR analysis was performed as described previously [21]. The primers for quantitative analysis of *Bcl*-*xL* and the housekeeping gene 60S acidic ribosomal proteins were: *Bcl*-*xL* (F) 5′- GATCCCCATGGCAGCAGTAAAGCAAG -3′ and (R) 5′- CCCCATCCCGGAAGAGTTCATTCACT -3′ and the house keeping gene 60S acidic ribosomal protein (F) 5′-ACGAGGTGTGCAAGGAGGGC-3′ and (R) 5′-GCAAGTCGTCTCCCATCTGC-3′.

### Western blot analysis

Cells and tissues were lysed and extracts were prepared as described previously [21]. Nuclear and cytoplasmic lysates were prepared with the CelLytic Nuclear Extraction Kit (Sigma-Aldrich) according to the manufacturer’s protocol. HIF-1α, p65 and Bcl-xL proteins in human cells were detected in 150 µg of cell extract using monoclonal anti-HIF-1α antibody (diluted 1:650; Novus), anti-p65 antibody (diluted 1:500; Novus) and anti-Bcl-xL antibody (1:600; Novus). Western blots were normalized using a monoclonal anti-β-actin antibody (diluted 1:10,000; Sigma-Aldrich) for cell extracts and a monoclonal anti- TATA box binding protein (TPB) (diluted 1:1,000; Sigma-Aldrich) for nuclear extracts.

### Reporter assays

The HIF-1α-luciferase reporter plasmid derived from our previous study [10] and NF-κB-luciferase reporter plasmid (Clontech) were utilized to determine HIF-1 and NF-κB-dependent transactivation of luciferase activities, respectively. In the measurement of HIF-1 and NF-κB-dependent transactivation of luciferase activities, the dual-luciferase reporter assay system (Promega) was used. U251 and U87 cells were transfected with each reporter construct and the TK-Renilla luciferase plasmid was used as a transfection control. Luciferase detection was performed 48 h after reporter construct transfection. Expression was calculated as the relative Firefly luciferase activity normalized with respect to the activity of transfection control Renilla luciferase. To determine the role of Tempol, YC-1 or Bay 11-7082 in cycling hypoxia-induced transcriptional activation of Bcl-xL, the stably Bcl-xL promoter-driven luciferase reporter-transfected U251 and U87 cells were incubated with Tempol (4 mM; Sigma-Aldrich), YC-1 (10 μM; Sigma-Aldrich) and Bay 11-7082 (5 μM; Sigma-Aldrich) together with in vitro cycling hypoxic stress for 4 h. Firefly luciferase activities were assayed and normalized to those of extract protein concentrations measured with the Bio-Rad protein assay kit (Bio-Rad). Luciferase activity was determined by mixing 10 μL of extracts from 1 × 10^5^ cells and 100 μL of luciferase assay reagent (Promega) according to the manufacturer’s instructions.

### ROS levels analysis

ROS levels were assessed by using carboxy-2′7′-dihydrodichlorofluorescein diacetate (H2DCFDA, Molecular Probes) to assess total ROS. Cells were incubated with 5 μg/mL of H2DCFDA for 30 min, then washed with PBS, trypsinized and collected in 1 mL of PBS. Fluorescent stained cells were transferred to polystyrene tubes with cell-strainer caps (Falcon) and subjected to FACScalibur instrument and FACSDiva 6.0 software (BD Bioscience) for acquisition and analysis.

### Vector constructions and viral transduction

The lentiviral vector pLKO AS2 (National RNAi Core Facility, Taiwan) was used as the backbone to generate a lentiviral reporter vector. The multiple cloning sites (MCS) of pTA-Luc vector (Clontech) was inserted with the cDNA fragment bearing −1075 to +617 *Bcl*-*xL* promoter to drive the expression of firefly luciferase gene. The *Bcl*-*xL* promoter driven reporter gene cassette was amplified from promoter to SV40 ploy A on the constructed pTA-Luc vector using PCR and inserted into pLKO AS2 as pLKO AS2- Bcl-xL-p by XhoI and MluI restriction enzymes. The pGreenFire1-SFFV [12] was used to generate glioblastoma reporter cells bearing SFFV promoter-driven a dual optical reporter gene encoding both green fluorescence protein (GFP) and luciferase (Luc). Lentiviral vectors carrying short hairpin RNAs (shRNA)-targeting HIF-1α (5′- TGCTCTTTGTGGTTGGATCTA-3′) and p65 (5′- CGGATTGAGGAGAAACGTAAA -3′) and scrambled shRNA (http://rnai.genmed.sinica.edu.tw/file/vector/C6-7/17.1.pLAS.Void.pdf) were provided by National RNAi core facility, Academia Sinica in Taiwan. The lentiviral vector pLVCT-tTR-KRAB (Addgene) was used to express Bcl-xL shRNA (Sigma-Aldrich) following the manufacturer’s protocol. Lentivirus production and cell transduction were carried out according to protocols described elsewhere [22, 23]. All constructs were confirmed by DNA sequencing. The U251, U87, GBM8401 cells bearing the SFFV promoter-driven a dual optical reporter gene and Bcl-xL promoter-driven Luc reporter gene were termed GBM8401/SFFV-LucGFP, U251- Bcl-xL-P-Luc and U87- Bcl-xL-P-Luc respectively.

### Fluorescence-activated cell-sorting (FACS) analyses

Tumor tissues were disaggregated with an enzyme cocktail containing collagenase type III (Sigma), hyaluronidase (Sigma), and collagenase type IV (Sigma), washed several times, and resuspended in phosphate-buffered saline (PBS) to produce a single cell suspension. Fluorescence was measured using a FACScalibur instrument and FACSDiva 6.0 software (BD Bioscience). Tumor cells were gated according to DsRed expression and side scatter (SSC). The hypoxic subpopulations were further gated or isolated based on the analysis of Hoechst 3342 and GFP fluorescence in dot plots. The control cells are derived from disaggregated the orthotopic GBM8401 or U87 xenografts, which are both Hoechst 3342 and GFP-negative, and were set in the lower left quadrant of the plot. Same setting conditions were used thereafter, cell populations located outside of this quadrant of the plot were defined as either Hoechst 3342^−^ and GFP^+^ cells (chronic hypoxic cells), Hoechst 3342^+^ and GFP^+^ cells (cycling hypoxic cells) or Hoechst 3342^+^ and GFP^−^ cells (Normoxic cells).

### Caspase-3 activity and apoptosis assays

U251 and U87 cells expressing Tet-inducible Bcl-xL shRNA were pretreated for 48 h with Dox (0.04 μg/mL) to induce Bcl-xL knockdown and then exposed to in vitro hypoxic stress, either non-interrupted hypoxic or cycling hypoxic stress, before TMZ treatment. The caspase-3-like protease activities were determined by the Caspase-3 Colorimetric Activity Assay Kit (Sigma-Aldrich) according to the manufacturer’s protocol. Briefly, Cell lysates were incubated with 2 mM Caspase-3 substrate (Ac-DEVD-pNA) in 1× assay buffer (20 mM HEPES, pH 7.4, 2 mM EDTA, 0.1 % CHAPS, 5 mM DTT) for 90 min at 37 °C. The absorbance was read at 405 nm and the results were calculated using a *p*-nitroaniline calibration curve. Annexin V and propidium iodide staining was performed to determine cell apoptosis using the Annexin V-FITC Apoptosis Detection Kit (Sigma-Aldrich) for 10 min at room temperature according to the manufacturer’s instructions, and then flow cytometric analysis was performed.

### Cytotoxicity assay

Cells were treated with DMSO (vehicle control), Tempol (4 mM; Sigma-Aldrich), TMZ (250 μM; Sigma-Aldrich) and the combination of Tempol and TMZ after in vitro hypoxic stress or cell sorting. After incubation at 37 °C for 48 h, the medium was removed from each well, 15 μL 3-94,5-dimethyl-2-yl-2,5-diphenyl-tetrazolium (MTT) (Sigma-Aldrich) solution (2 mg/mL) were added and the plates were incubated at 37 °C for 4 h. The reaction was stopped by the addition of 100 μL of isopropanol/HCl, and the absorbance at 570 nm was recorded on a μQuant plate reader (Bio-Tek).

### Animal models

Eight-week-old male athymic nu/nu mice were used to establish animal tumor models. For the subcutaneous GBM xenograft model, 5 × 10^6^ GBM8401- Bcl-xL-P-Luc cells were injected subcutaneously into the dorsal flank and small (80 ± 16.0 mm^3^) subcutaneous tumors developed 14 days later were used for animal imaging studies. For the orthotopic GBM xenograft model, 2 × 10^5^ GBM8401/SFFV-LucGFP cells were harvested by trypsinization and injected into the right basal ganglia of anesthetized mice. The tumors developed at 12 days after tumor implantation for evaluating the efficiency of therapy studies. All animal studies were conducted according to the Institutional Guidelines of China Medical University and approved by the Institutional Animal Care and Use Committees of China Medical University (approval number 102-54-N).

### In vivo treatment

For the bioluminescence imaging of in vivo transcriptional activation of Bcl-xL,

Mice bearing U87- Bcl-xL-P-Luc xenograft were received with vehicle, Tempol (250 mg/kg i.p.), YC-1 (15 mg/kg i.p.) or Bay 11-7082 (10 mg/kg i.p.) followed by in vivo cycling hypoxic treatment. The procedure for in vivo cycling hypoxic treatment was carried out following published methods [14, 24]. Briefly, the tumor-bearing mice were exposed to continuous flow of a humidified gas mixture to induce in vivo hypoxia in 6-liter hypoxia chambers. The mice were exposed to normal air (control) or 12 cycles of 10 min 7 % O_2_ breathing interrupted by 10 min periods of normal air breathing for cycling hypoxic treatment. For the therapeutic study, intracerebral gioblastoma-bearing mice were randomly assigned to four different therapeutic groups: control (vehicle treatment), pretreatment of Tempol (250 mg/kg i.p.), TMZ (5 mg/kg i.p.) or pretreatment of Tempol (250 mg/kg i.p.) + TMZ (5 mg/kg i.p.). The pretreatment of Tempol was performed at day 13 after tumor cell injection. Systemic TMZ treatment was performed for 5 days, starting on day 14 after tumor cell injection. Tumor progression was monitored by bioluminescence imaging and mice were monitored daily for survival. Animals were killed at the onset of neurologic signs or any type of distress.

### Bioluminescent imaging (BLI)

Mice were imaged with the IVIS Imaging System 200 Series (Caliper) to record bioluminescent signal emitted from the engrafted tumors. Mice were anesthetized with isoflurane and received intraperitoneal injection of D-Luciferin (Caliper) at a dose of 270 µg/g body weight. Imaging acquisition was performed at 15 min after intraperitoneal injection of luciferin. For BLI analysis, regions of interest encompassing the intracranial area of signal were defined using Living Image software, and the total number of photons per second per steradian per square centimeter were recorded. To facilitate comparison of growth rates, each mouse’s luminescence readings were normalized against its own luminescence reading at day 12, thereby allowing each mouse to serve as its own control.

### Statistical analysis

One-way analysis of variance with post hoc Scheffe analyses and Kaplan–Meier Survival Analysis with Tarone-Ware statistics were carried out using the SPSS package (version 18.0). The differences between control and experimental groups were determined by the two-sided, unpaired Student t test. *P* < 0.05 was considered significant.

## Results

### ROS is required for cycling hypoxia-mediated HIF-1α and NF-κB activation

**Fig. 1:**
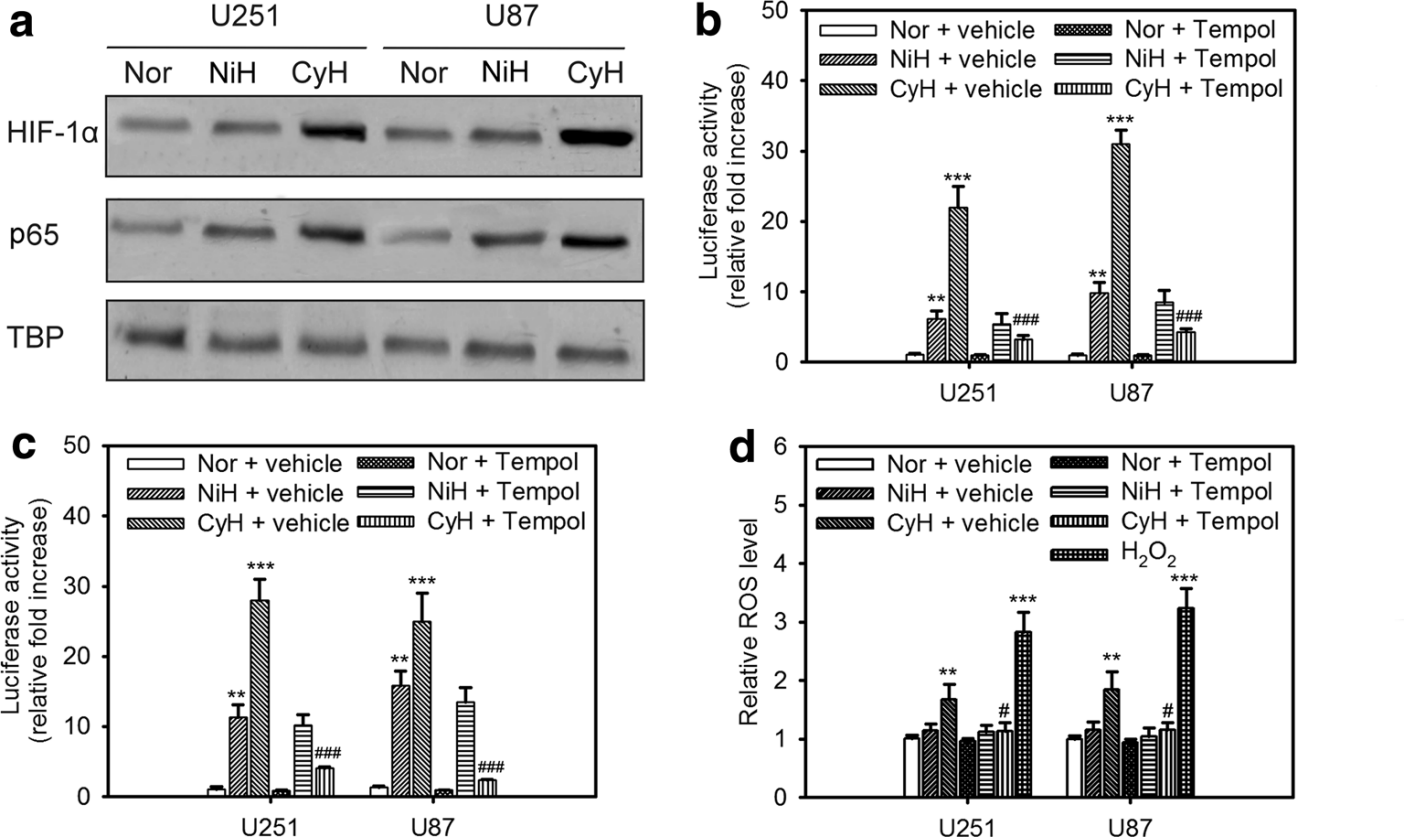
ROS is required for cycling hypoxia-mediated HIF-1α and NF-κB activation. The levels of nuclear HIF-1α and p65 (**a**), HIF-1 dependent transactivation of luciferase activity (**b**), NF-κB-dependent transactivation of luciferase activity (**c**), and intracellular ROS (**d**) in U251and U87 glioblastoma cells exposed to normoxia (Nor), uninterrupted hypoxia (NiH), or cycling hypoxia (CyH) (<1% O_2_) with or without Tempol. TATA-binding protein (TBP) was used to normalize protein loading for nuclear extracts. *Error bars* denote the standard deviation within triplicate experiments. **P < 0.01, ***P < 0.001 compared to normoxia. ^#^P < 0.05, ^###^P < 0.001 compared to vehicle treatment

Our previous studies indicated that NADPH oxidase subunit 4-mediated ROS contributes to the cycling hypoxia-induced HIF-1α activation in glioblastoma cells [12–14]. However, the role of cycling hypoxia in NF-κB signaling is still unclear. Therefore, we examined the effect of experimentally imposed uninterrupted or cycling hypoxic stress on HIF-1α and NF-κB activation in glioblastoma cells. To assess HIF-1α and NF-κB activation under uninterrupted or cycling hypoxia in glioblastoma cells, the amount of HIF-1α and NF-κB protein in nuclear extracts was determined by western blot analysis after cells were exposed to hypoxic treatment in vitro. Under both uninterrupted and cycling hypoxic stress, HIF-1α and NF-κB protein levels increased in U87 or U251 glioblastoma cell nuclei (Fig. 1a). However, both HIF-1α and NF-κB protein levels in glioblastoma cells under cycling hypoxia stress were higher than those in cells under uninterrupted hypoxia stress. Moreover, reporter assays also showed that this effect further resulted in differences in HIF-1α and NF-κB signal transduction activities. As shown in Fig. 1b, c, both HIF-1α and NF-κB signal transduction activities in the cycling hypoxia-treated cells were significantly increased after treatment in response to higher HIF-1α or NF-κB protein accumulation in cell nuclei. Next, we determined whether cycling hypoxia induced ROS contributes to HIF-1α and NF-κB activation. The glioblastoma cells were treated with Tempol during cycling hypoxia. When Tempol was administered following cycling hypoxia, the cycling hypoxia-induced H2DCF-DA response was completely suppressed (Fig. 1d). Furthermore, the increase in HIF-1α and NF-κB activation triggered by cycling hypoxia was abrogated (Fig. 1b, c). Taken together, these results indicate that cycling hypoxia promotes HIF-1α and NF-κB activation in glioblastoma cells via ROS.

### Cycling hypoxia mediates Bcl-xL expression via HIF-1α or NF-κB activation

**Fig. 2:**
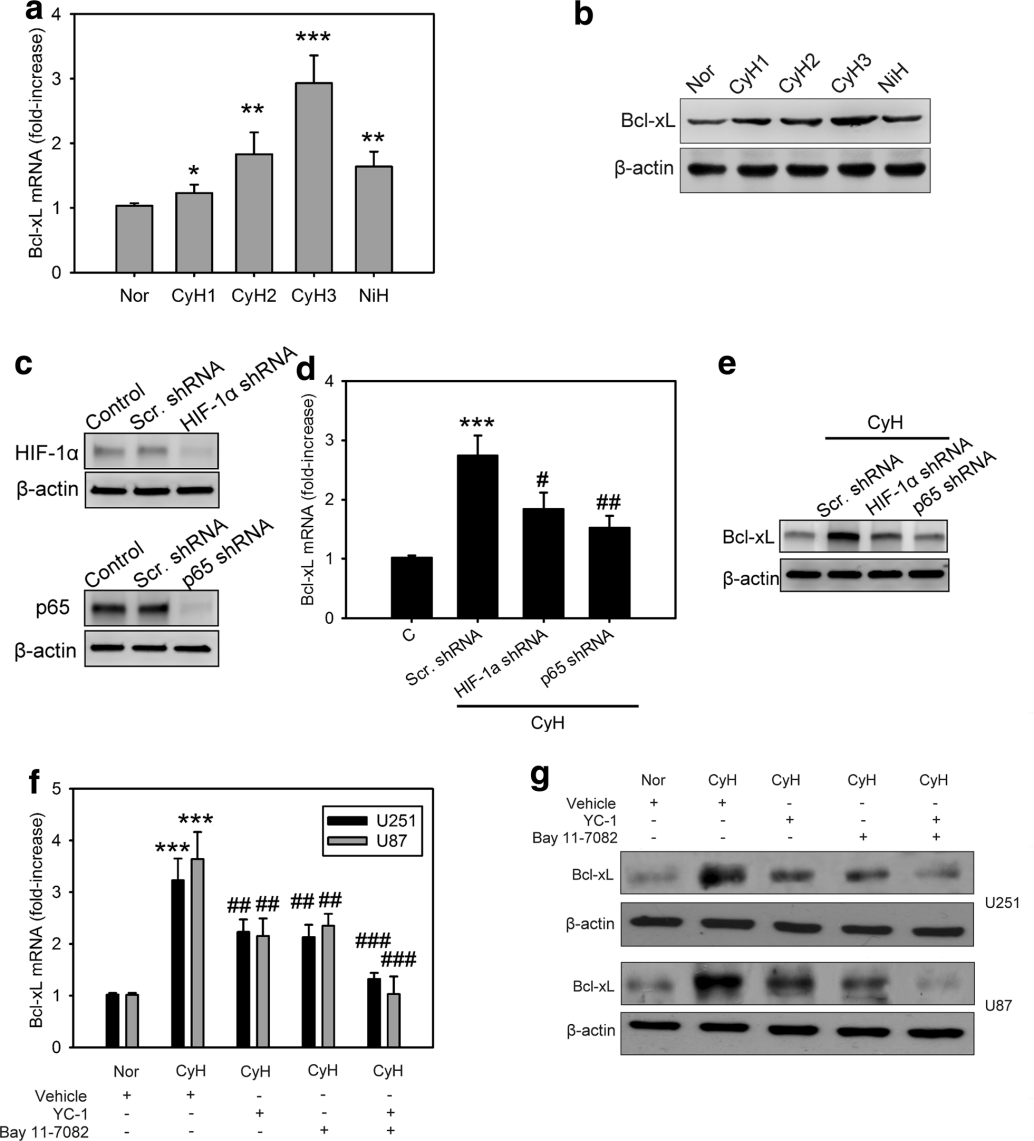
Cycling hypoxia mediates Bcl-xL expression via HIF-1α or NF-κB activation. Bcl-xL mRNA (**a**) and protein (**b**) levels in GBM8401 glioblastoma cells collected before and after 1 h of each of the 3 cycles of hypoxia (<1 % O_2_) during cycling hypoxia (CyH) and after 4 h of uninterrupted hypoxia (NiH). *P < 0.05, **P < 0.01, ***P < 0.001 compared to normoxia. **c** Western blot analysis of HIF-1α or p65 knockdown in GBM8401 glioblastoma cells via the lentiviral-based HIF-1α or p65 shRNA. **d**, **e** Bcl-xL mRNA and protein levels in GBM8401 glioblastoma cells transfected with or without scramble (Scr.), HIF-1α, or p65 shRNA for 48 h followed by stimulation with cycling hypoxic stress for 4 h. ***P < 0.001 compared to normoxia (**c**). ^#^P < 0.01, ^##^P < 0.001 compared to Scr. shRNA treatment. **f**, **g** Bcl-xL mRNA and protein levels in U251 and U87 cells treated with cycling hypoxic stress for 4 h in the absence or presence of HIF-1α (YC-1) and NF-κB (Bay 11-7082) inhibitors. *Error bars* denote the standard deviation within triplicate experiments. ***P < 0.001 compared to normoxia (Nor). ^##^P < 0.01, ^###^P < 0.001 compared to vehicle treatment

It was previously reported that HIF-1α or NF-κB activation up-regulates the expression of Bcl-xL, thereby preventing cell death at the mitochondrial level [20, 25]. Therefore, we next examined whether the regulation of Bcl-xL in glioblastoma cells was affected by cycling hypoxia-induced HIF-1α and NF-κB activation. We first evaluated the abundance of Bcl-xL at different times in the hypoxia-reoxygenation protocol in GBM8401 cells. Bcl-xL expression level was increased after each new cycle of hypoxia treatment (Fig. 2a, b). Bcl-xL mRNA and protein levels in GBM8401 cells under cycling hypoxia stress were significantly higher than those in GBM8401 cells under uninterrupted hypoxia stress. In an attempt to gain insights into the role of HIF-1α or NF-κB on the regulation of Bcl-xL, we examined Bcl-xL expression in GMB8401cells lacking HIF-1α or NF-κB expression. Lentiviral transduction with shRNAs against HIF-1α or NF-κB resulted in the loss of HIF-1α or NF-κB expression in GBM8401 cells when compared with that in cells transduced with scrambled shRNA (Fig. 2c). HIF-1α or NF-κB knockdown in GBM8401 cells significantly inhibited cycling hypoxia-induced Bcl-xL expression (Fig. 2d, e). Moreover, glioblastoma cells, U87 and U251, were treated with specific HIF-1α (YC-1) and NF-κB (Bay 11-7082) inhibitors during cycling hypoxic treatment. Bcl-xL mRNA and protein levels were upregulated after cycling hypoxia, but these effects were inhibited by YC-1 and Bay 11-7082 treatment, suggesting that HIF-1α and NF-κB are critical signaling molecules involved in cycling hypoxia-induced Bcl-xL expression (Fig. 2f, g).

### Cycling hypoxia-mediated Bcl-xL expression results in chemoresistance

**Fig. 3:**
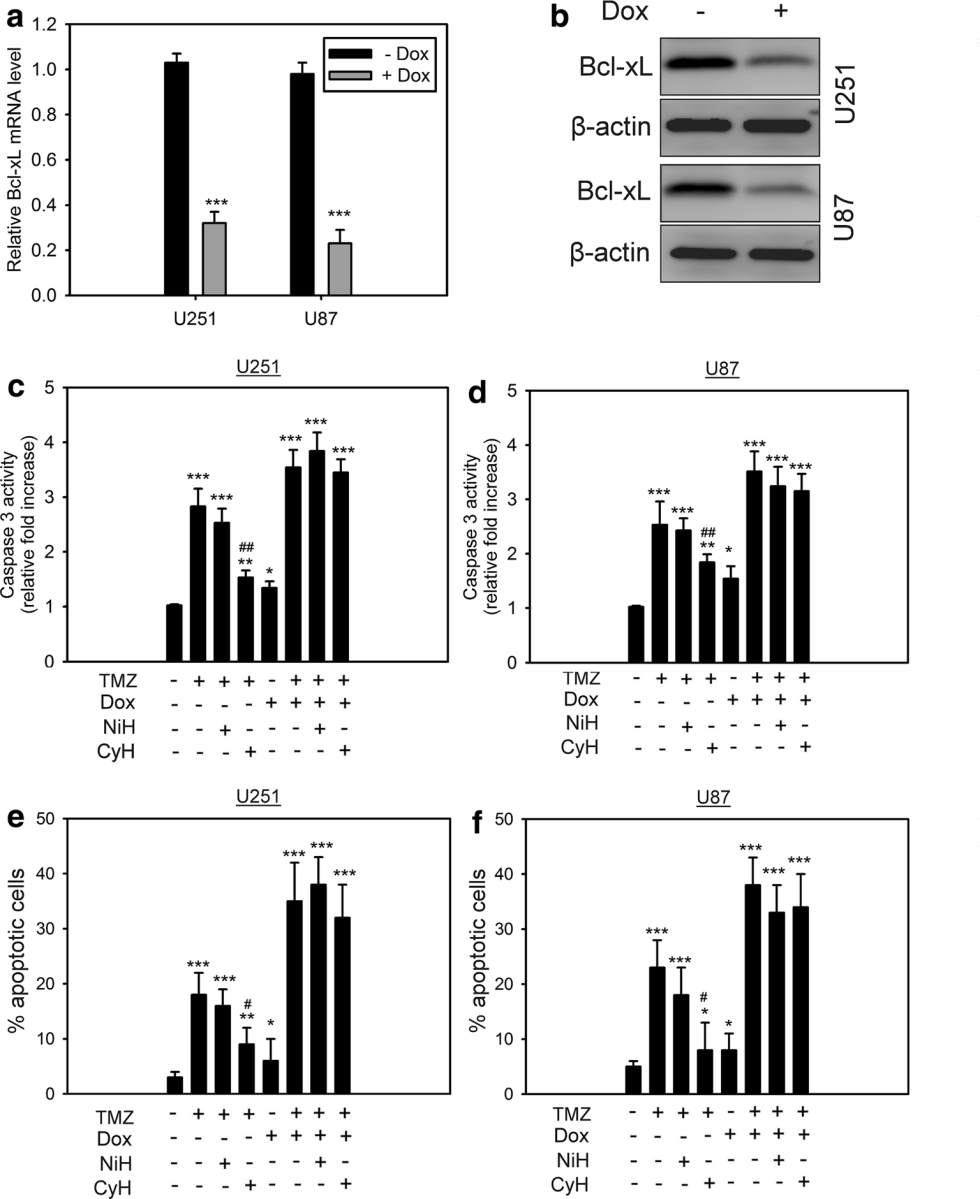
Cycling hypoxia-mediated Bcl-xL expression results in chemoresistance. **a** Verification of Bcl-xL knockdown by Tet-regulatable lentiviral knockdown system at the mRNA (**a**) and protein (**b**) levels. The lentiviral infected cells were treated for 48 h with Dox (0.04 μg/mL) to induce Bcl-xL knockdown. *Error bars* denote the standard deviation among triplicate experiments. *P < 0.05, **P < 0.01, ***P < 0.001 compared to no Dox treatment. Caspase-3 activity (**c**, **d**) and percentage of apoptotic cells (**e**, **f**) in U251 and U87 cells cultured in normoxia, uninterrupted (NiH) hypoxia (<1 % O_2_), and cycling (CyH) hypoxia (<1 % O_2_) with or without Bcl-xL knockdown in response to temozolomide (TMZ) treatment. Cells were pretreated for 48 h with Dox (0.04 μg/mL) to induce Bcl-xL knockdown and exposed to hypoxic stresses before TMZ treatment. *Error bars* denote the standard deviation among triplicate experiments. *P < 0.05, **P < 0.01, ***P < 0.001 compared to control without any treatment. ^#^P < 0.05, ^##^P < 0.01 compared to normoxia with TMZ treatment

Given the recognized role of Bcl-xL as an apoptosis suppressor, we determined whether cycling hypoxia-mediated Bcl-xL up-regulation in glioblastoma cells was involved in the cell resistance to chemotherapy. Temozolomide (TMZ) was chosen as the chemotherapy drug, because this drug has been widely used in the treatment of patients with glioblastoma [26]. The apoptotic changes were confirmed by detecting caspase activities and apoptotic cells via the fluorogenic caspase-3 substrate and apoptosis assay, respectively. To investigate the role of Bcl-xL in hypoxia-mediated anti-apoptosis, Tet-regulatable lentiviral vectors encoding shRNAs were used to stably and specifically knockdown Bcl-xL induction in U251 and U87 cells under hypoxia. Q-PCR and western blot analysis showed that this shRNA successfully knocked down Bcl-xL expression in both cell lines after doxycycline (Dox) treatment (Fig. 3a, b). We first used these cells to examine the effect of in vitro cycling hypoxic or uninterrupted hypoxic stress on TMZ-induced caspase activities and apoptosis in glioblastoma cells. Our results demonstrated that caspase activity and the number of apoptotic cells were significantly decreased by cycling hypoxia compared to those in normoxic cells or uninterrupted hypoxia-treated cells (Fig. 3c–f), suggesting that cycling hypoxic cells are resistant to TMZ-induced apoptosis. However, after Dox pretreatment, cycling hypoxia-induced chemoresistance was inhibited by Bcl-XL knockdown. These results indicate that Bcl-XL plays an important role in cycling hypoxia-induced chemoresistance in glioblastoma cells.

### Superoxide dismutase mimetic, Tempol, inhibits cycling hypoxia-induced chemoresistance

**Fig. 4:**
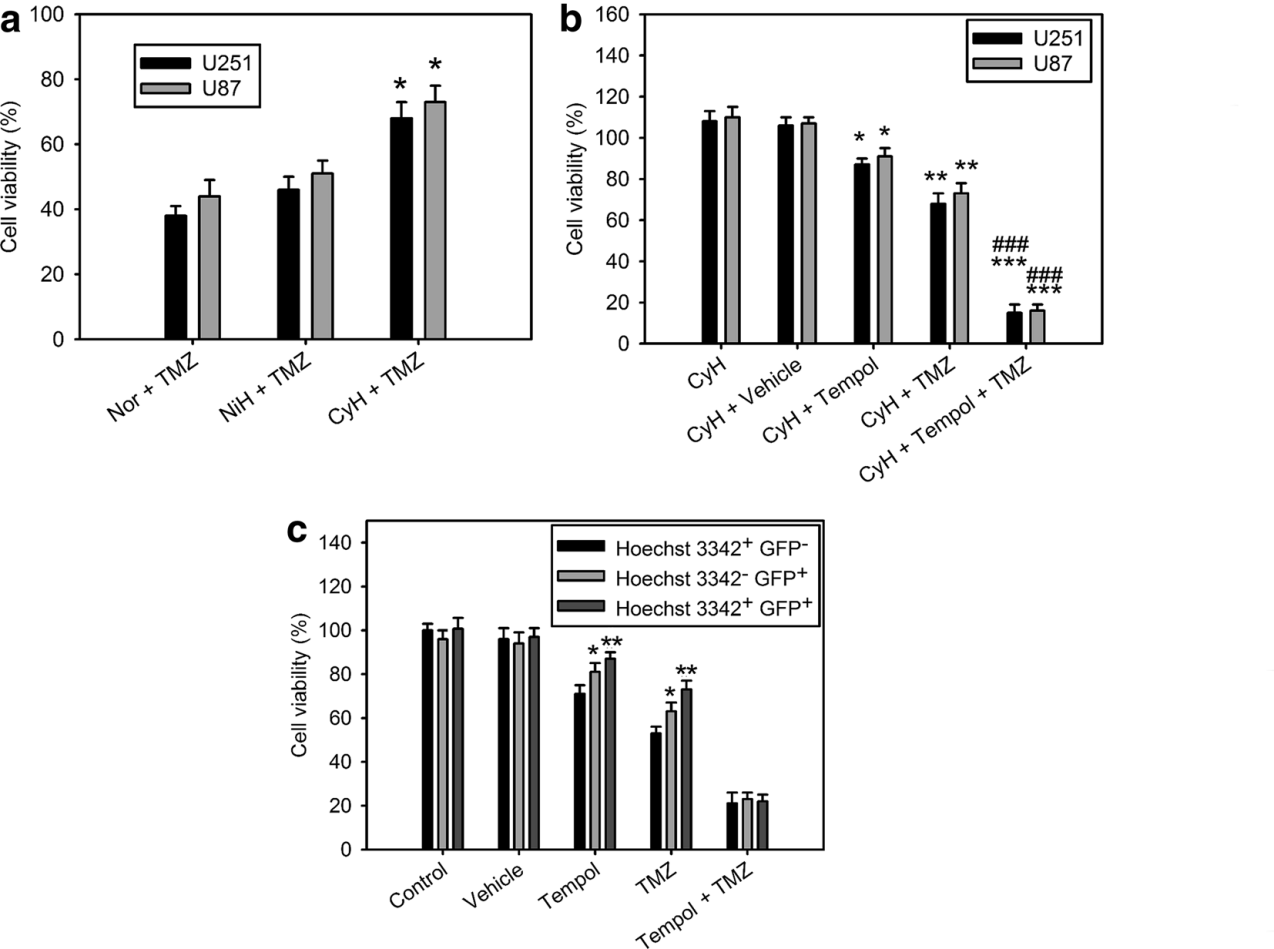
Superoxide dismutase mimetic, Tempol, inhibits cycling hypoxia-induced chemoresistance. **a** Cytotoxicity assay of normoxia (Nor), uninterrupted hypoxia (NiH), and cycling hypoxia (CyH)-mediated temozolomide (TMZ) sensitivity in U251 and U87 glioblastoma cells. *P < 0.05 compared to Nor with TMZ treatment. **b** Cytotoxicity assay after Tempol treatment revealed increased TMZ cytotoxicity and suppression of cycling hypoxia (CyH)-mediated TMZ resistance in U251 and U87 glioblastoma cells. *P < 0.05, **P < 0.01, ***P < 0.001 compared to CyH without any treatment, ^###^P < 0.001 compared to CyH with TMZ treatment. **c** Cytotoxicity assay of TMZ in normoxic tumor cells (Hoechst 3342^+^ and GFP^−^), chronic hypoxic tumor cells (Hoechst 3342^−^ and GFP^+^), and cycling hypoxic tumor cells (Hoechst 3342^+^ and GFP^+^) isolated from disaggregated U87/hif-1-r xenografts. *P < 0.05, **P < 0.01 compared to normoxic tumor cells (Hoechst 3342^+^ and GFP^−^). *Error bars* denote the standard deviation among triplicate experiments

The above findings suggest that cycling-induced Bcl-xL expression contributes the chemoresistance via ROS-mediated HIF-1α and NF-κB activation. We next tested whether ROS blockage can improve cycling hypoxia-induced chemoresistance. U251 and U87 cells were treated with Tempol under cycling hypoxia followed by TMZ treatment. MTT assays were used to determine TMZ cytotoxicity in glioblastoma cells. Cycling hypoxia significantly increased glioblastoma cell chemoresistance to TMZ compared to that of normoxic controls (Fig. 4a). When ROS was blocked by Tempol, the chemoresistance induced by cycling hypoxic stress decreased (Fig. 4b). To further determine the drug sensitivities of hypoxic cell subpopulations in glioblastoma xenografts, Hoechst 33342 staining and HIF-1 activation labeling together with immunofluorescence imaging and fluorescence-activated cell sorting were utilized to isolate hypoxic tumor subpopulations from human glioblastoma xenografts as described in our previous studies [6, 13, 14]. The chemoresistance increased significantly in cycling hypoxic cells (Hoechst 3342^+^ and GFP^+^) and chronic hypoxic cells (Hoechst 3342^−^ and GFP^+^), compared with that in normoxic cells (Hoechst 3342^+^ and GFP^−^) (Fig. 4c). Moreover, treatment with Tempol significantly enhanced the TMZ-mediated cytotoxicity in cycling and chronic hypoxic cells, suggesting that the superoxide dismutase mimetic is able to improve tumor hypoxia-induced chemoresistance.

### Tempol inhibits cycling hypoxia-induced Bcl-xL induction and expression in vivo

**Fig. 5:**
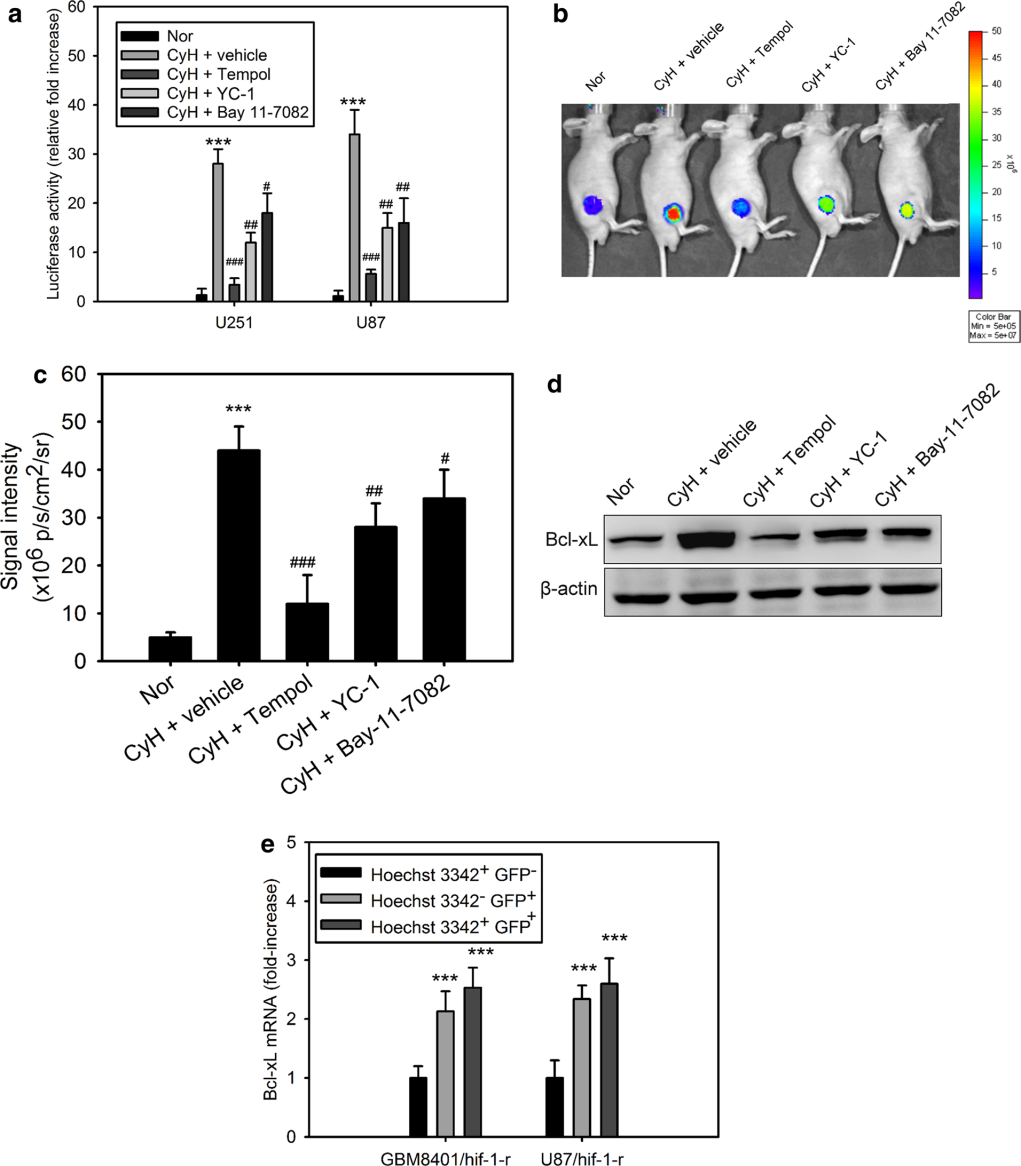
Tempol inhibits cycling hypoxia-induced Bcl-xL induction and expression in vivo. **a** Bcl-xL promoter-dependent transactivation of luciferase activity in U251 and U87 cells cultured in normoxia (Nor) and cycling (CyH) hypoxia (<1 % O_2_) with or without pretreatment with Tempol, YC-1, or Bay 11-7082. ***P < 0.001 compared to Nor. ^#^P < 0.05, ^##^P < 0.01, ^###^P < 0.001 compared to CyH with vehicle treatment. **b** In vivo bioluminescence (BLI) images of the transcriptional activation of Bcl-xL in U87 glioblastoma xenografts before and after Tempol, YC-1, or Bay 11-7082 treatment. **c** Quantitative data obtained from the BLI imaging of the transcriptional activation of Bcl-xL in U87 xenografts. Data represent the mean ± standard deviation of average counts within the tumor region of interest in BLI from 6 mice. ***P < 0.001 compared to Nor. ^#^P < 0.05, ^##^P < 0.01, ^###^P < 0.001 compared to CyH with vehicle treatment. **d** Bcl-xL protein levels in homogenized tumor tissues derived from the above groups. **e** *Bcl*-*xL* mRNA levels in normoxic cells (Hoechst 3342^+^ and GFP^−^), chronic hypoxic cells (Hoechst 3342^−^ and GFP^+^), and cycling hypoxic cells (Hoechst 3342^+^ and GFP^+^) isolated from disaggregated GBM8401/hif-1-r and U87/hif-1-r xenografts. ***P < 0.001 compared to normoxic tumor cells (Hoechst 3342^+^ and GFP^−^). *Error bars* denote the standard deviation within triplicate experiments

We examined the role of ROS in cycling or uninterrupted hypoxia-mediated Bcl-xL induction. We first verified whether the transcriptional activation is involved in hypoxia-mediated Bcl-xL induction in glioblastoma cells. To achieve this, U87 and U251 glioblastoma cells were stably transfected with a lentiviral vector bearing the Bcl-xL promoter −1075 to +617 region driven luciferase reporter gene that allowed for dynamic monitoring of the transcriptional activation of Bcl-xL. As shown in Fig. 5a, the transcriptional activation of Bcl-xL in the cycling hypoxia-treated cells was significantly increased after in vitro cycling hypoxic treatment and this effect was inhibited by Tempol, YC-1, and Bay 11-7082. We next sought to investigate whether the effects observed in vitro were also detected in tumor-bearing mice. U87-Bcl-xL-P-Luc reporter cells were implanted subcutaneously into the flank of nude mice. The tumors developed 14 days later. *In vivo* optical imaging was used to record reporter activity in these tumors following in vivo cycling hypoxic treatment. The 7 % O_2_ breathing conditions resulted in a tumor pO_2_ of <2–3 mm Hg. In vivo optical imaging demonstrated that significantly enhanced levels of luciferase activity were detectable in the animals subjected to cycling hypoxic stress (Fig. 5b, c). However, cycling hypoxia-induced transcriptional activation of Bcl-xL was inhibited in mice treated with Tempol, YC-1, and Bay 11-7082 followed by in vivo cycling hypoxic treatment (Fig. 5b, c). Western blotting of homogenized tumor tissues confirmed the effect on Bcl-xL protein expression levels (Fig. 5d). Besides, the total RNA of the hypoxic cell subpopulations derived from disaggregated orthotopic GBM8401/hif-1-r and U87/hif-1-r xenografts [6] were further analyzed for *Bcl*-*xL* expression by Q-PCR. *Bcl*-*xL* expression significantly increased in cycling and chronic hypoxic cells compared with that in normoxic cells (Fig. 5e). These data indicated that cycling hypoxic stress resulted in significantly elevated levels of *Bcl*-*xL* induction in glioblastoma cells and xenografts. ROS and their downstream transcription factors, HIF-1α and NF-κB, are critical mediators involved in cycling hypoxia-mediated *Bcl*-*xL* induction.

### Tempol enhances the efficiency of TMZ chemotherapy in mice bearing intracranial glioblastoma xenografts

**Fig. 6:**
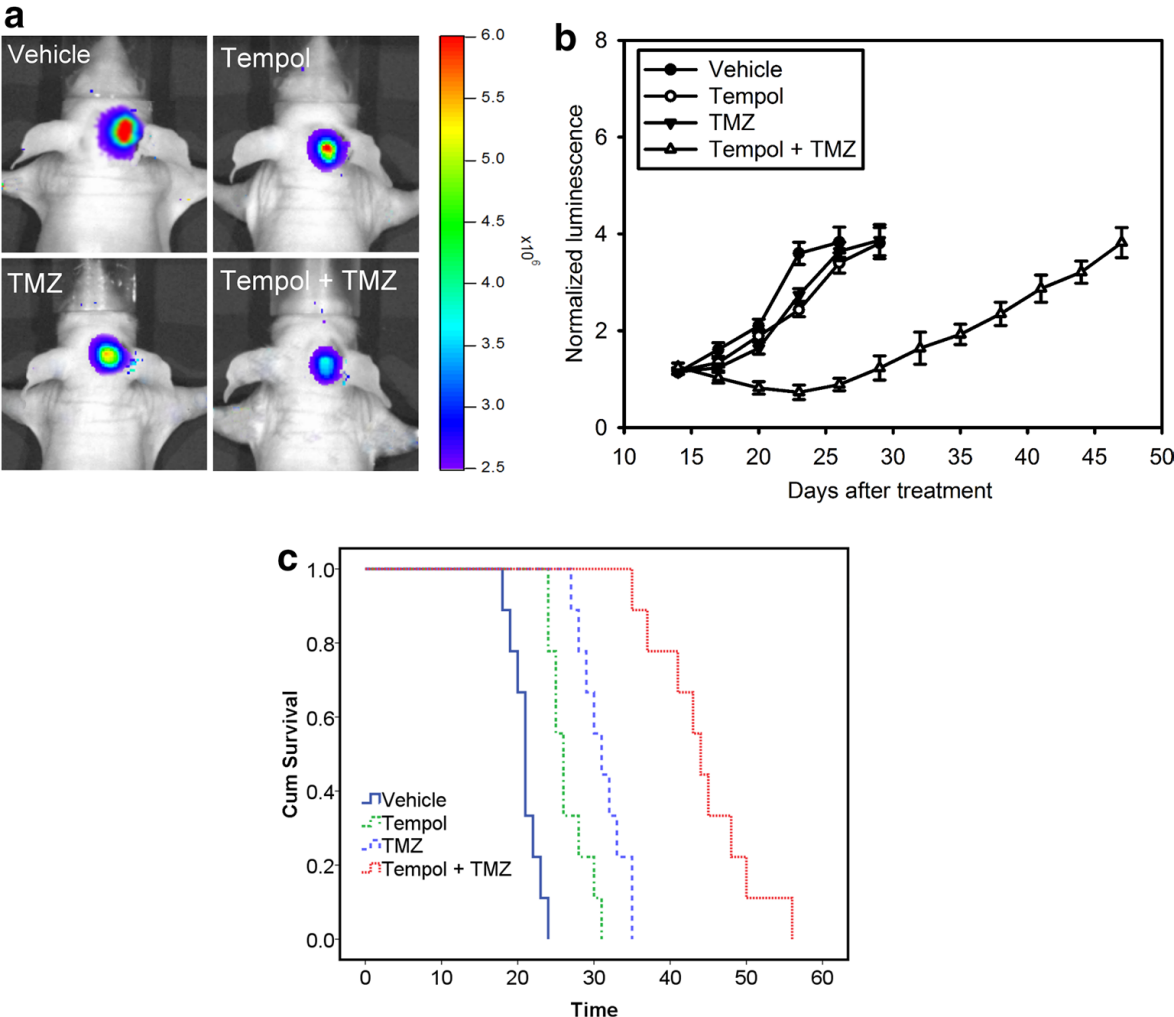
Tempol enhances the efficiency of TMZ chemotherapy in mice bearing intracranial glioblastoma xenografts. **a** Bioluminescent images from control (vehicle) and animals treated with Tempol, TMZ, and Tempol plus TMZ on day 20 after tumor implantation. **b** The mean normalized BLI values associated with longitudinal monitoring of intracranial tumor growth for each treatment group. *Error bars* denote the standard deviation among 9 mice per group. **c** The corresponding survival curves of GBM8401 xenograft-bearing mice for each treatment group

Finally, we investigated whether ROS scavenger represents a viable approach to inhibit tumor microenvironment-mediated therapy resistance in GBM. We sought to test the efficacy of Tempol in combination with TMZ treatment. BLI was utilized to assess intracranial tumor growth in the orthotopic GBM8401/SFFV-LucGFP xenograft model. Consistent with the in vitro studies, mice treated with Tempol prior to initiation of the TMZ treatment presented with a significantly increased tumor growth delay and survival rate than those pretreated with vehicle (Fig. 6a–c). The Tempol and TMZ combination treatment yielded synergistic effects. These results suggest that ROS scavenger enhances the efficiency of TMZ chemotherapy in vivo in orthotopic glioblastoma xenografts.

## Discussion

There is abundant evidence to suggest that cycling hypoxia as well as uninterrupted hypoxia play roles in many aspects of tumor growth and development [27, 28]. However, some results demonstrated that cycling hypoxia would utilize different intracellular signaling pathways than those utilized by uninterrupted hypoxia. For instance, ROS might play a vital role in cycling hypoxia-induced alterations in the carotid body function [29]. In addition, the expression of immediate early response genes and transcription factors, AP-1 and HIF-1, were activated by cycling hypoxia [29]. These results suggest that signaling pathways associated with transcriptional regulation by cycling hypoxia are distinct from those utilized by uninterrupted hypoxia. Consistent with these findings, our data showed that ROS production is only significantly triggered by cycling hypoxia in glioblastoma cells. Therefore, the ROS-mediated HIF-1/NF-κB/Bcl-xL pathway is activated only in cycling hypoxia-treated glioblastoma cells. Furthermore, cycling hypoxia induced more chemoresistance than uninterrupted hypoxia in U87 or U251 glioblastoma cells.

Highly aggressive tumors are known to be exposed to hypoxia, which occurs as a result of inadequate blood supply [30]. Since hypoxia can affect certain gene regulatory mechanisms and signal transduction pathways, including tumor cell apoptosis, metastasis, and tumor angiogenesis, deciphering the hypoxic tumor cell response is essential to understand tumor progression. HIF-1α is a master transcription factor that regulates both physiological and pathophysiological responses of mammalian cells to hypoxia [31]. In addition, it is also regulated by other factors, including oncogenes, growth factors, and free radicals [32]. It is well known that HIF-1α overexpression driven by hypoxia and free radical species contributes to therapy resistance [2, 10]. Several signaling pathways have been proposed to be regulated by hypoxia. For example, hypoxia could activate the JNK and p38 stress kinases in human squamous carcinoma cells and further leads to phosphorylation of transcription factor ATF-2 [33]. Moreover, HIF-1α signaling associated with p38 involves mitochondrial-derived ROS [34]. Furthermore, we previously demonstrated that cycling hypoxia induces HIF-1 activity via ROS-mediated HIF-1α synthesis and stabilization in U87 glioblastoma cells [10].

Constitutive activation of NF-κB frequently occurs in various cancer types and is important for cancer cells to escape apoptosis and survive in the presence of apoptotic stimuli [35]. Therefore, cancer cell resistance to chemotherapeutic agents can be partly explained by NF-κB deregulation. Inhibition of NF-κB activity could sensitize hepatocellular carcinoma cells to doxorubicin-induced apoptosis [36]. In addition, NF-κB is activated by hypoxia and plays a central role in gene regulation under hypoxia/reoxygenation [37, 38]. However, to date, the involvement of ROS in NF-κB signaling is controversial. For example, some reports suggested that NF-κB is not a sensor of oxidative stress and ROS does not influence NF-κB activation during intermittent hypoxia/reoxygenation [39, 40]. In contrast, our results show that cycling hypoxia induces HIF-1α and NF-κB activation through ROS generation and this activation dramatically decreases after Tempol treatment in both U87 and U251 glioblastoma cells. A recent review article also highlights that ROS have various inhibitory or stimulatory roles in NF-κB signaling, suggesting the complexity of ROS-mediated NF-κB signaling [41].

GBM is the most malignant form of brain cancer with a high mortality rate and this aggressive capability may be partly due to tumor hypoxia [42, 43]. It contains multiple hypoxic areas that exhibit elevated HIF-1 signal transduction activity, resulting in the deregulated expression of downstream target genes that contribute to GBM malignancy [44]. The expression of BNIP3, a Bcl-2 family member, correlates with HIF-1α expression levels in various types of tumor cells. Its expression was predominantly detected in the nucleus under hypoxic stress and contributes to cell survival in GBM [45]. The Bcl-xL and Bcl-2 proteins are dominant inhibitors of apoptotic cell death. Recent studies demonstrated that Bcl-xL is one of the downstream target genes of HIF-1α and NF-κB through transcriptional regulation [19, 20, 25]. Therefore, we proposed that the activation of HIF-1α and NF-κB induced by ROS under cycling hypoxia could result in Bcl-xL induction and allow the survival of glioblastoma cells. Our results clearly indicate that cycling hypoxia induces Bcl-xL expression in vivo and in vitro via HIF-1α or NF-κB activation and these effects can be inhibited by Tempol, HIF-1α inhibitor, or NF-κB inhibitor treatment. ROS and their downstream transcription factors, HIF-1α and NF-κB, are critical mediators involved in cycling hypoxia-mediated Bcl-xL induction. In addition, unlike uninterrupted hypoxia treatment, cycling hypoxia induces prosurvival effects in glioblastoma cells in response to Bcl-xL induction and caspase-3 inhibition. Moreover, cycling hypoxia significantly increases chemoresistance to TMZ compared with normoxic conditions in U87 or U251 glioblastoma cells. This chemoresistance induced by cycling hypoxic stress was suppressed by a ROS scavenger as well as by HIF-1α and NF-κB inhibitors. Therefore, ROS mediated HIF-1α and NF-κB activation is a crucial mechanism involved in cycling hypoxia-induced anti-apoptosis and chemoresistance in glioblastoma cells. These findings are important in the selection of optimal strategies for anticancer therapy in GBM.

In conclusion, the present study provides insightful information regarding the differential regulatory mechanisms involved in cycling hypoxia and uninterrupted hypoxia in tumor chemosensitivity. In addition, our data also highlight the putative mechanisms of cycling hypoxia in tumor cell chemoresistance in glioblastoma and suggest that ROS are attractive therapeutic targets to counteract cycling hypoxia-induced chemoresistance. Our previous studies also suggests that ROS within GBM cells act as second messengers in intracellular signaling cascades, which contribute to cycling hypoxia-mediated tumor progression [12, 14]. Since cycling hypoxia is now a well-recognized phenomenon in the tumor microenvironment [2], ROS blockade should be used before and with chemotherapy to suppress cycling hypoxia-induced chemoresistance and tumor progression and to further enhance the therapeutic efficiency of cytotoxic therapies in GBM.

## Abbreviations

Bcl-xL: B-cell lymphoma extra-long
GBM: glioblastoma multiforme
HIF-1α: hypoxia-inducible factor-1 alpha
HRE: hypoxia-responsive element
MDR: multidrug resistance
NF-κB: nuclear factor-κb
Nox4: NADPH oxidase subunit 4
ROS: reactive oxygen species
TMZ: temozolomide
